# O010. Migraine aura symptoms last for more than one hour in more than one quarter of patients: results from a prospective diary-aided study

**DOI:** 10.1186/1129-2377-16-S1-A65

**Published:** 2015-09-28

**Authors:** Michele Viana, Mattias Linde, Grazia Sances, Natascia Ghiotto, Elena Guaschino, Marta Allena, Giuseppe Nappi, Peter J Goadsby, Cristina Tassorelli

**Affiliations:** Headache Science Centre, C. Mondino National Neurological Institute, Pavia, Italy; Department of Neuroscience, Norwegian University of Science and Technology, Trondheim, Norway; Norwegian Advisory Unit on Headaches, St. Olavs University Hospital, Trondheim, Norway; Headache Group, NIHR-Wellcome Trust Clinical Research Facility, King's College, London, UK; Department of Brain and Behavioural Sciences, University of Pavia, Pavia, Italy

## Background

As there are no biological markers, a detailed description of symptoms, particularly temporal characteristics, is crucial when diagnosing migraine aura. In ICHD-IIIbeta, migraine aura duration is considered normal when each symptom is no longer than one hour. A recent systematic review of the topic[1] did not find any article exclusively focusing on the duration of the aura. The pooled analysis of data from the literature on aura duration showed that visual symptoms lasting for more than one hour occurred in 6%-10% of patients, sensory symptoms in 14%-27% and dysphasic aura in 17%-60%. Here we investigated the duration of aura symptoms, using a prospective diary-aided approach.

## Methods

We recruited 176 consecutive patients affected by migraine with aura at the Headache Centres of Pavia and Trondheim. The study received approval by the local Ethics Committees. All patients signed an informed consent form. All the patients prospectively recorded the characteristics of three consecutive attacks in an *ad hoc* aura diary that included the time of onset and the end of each aura symptom and the headache.

## Results

Fifty-four patients completed the study recording in a diary the characteristics of three consecutive auras (n=162 auras). Out of 162 auras that were evaluated, visual symptoms occurred in 159 (97%), sensory symptoms in 52 (32%), and dysphasic symptoms in 18 (11%). The cumulative number of aura symptoms recorded was therefore 229. The median duration of visual, sensory and dysphasic symptoms was 30, 20 and 20 minutes, respectively. Visual symptoms lasted for more than one hour in 14% of auras (n=158), sensory symptoms in 21% of auras (n=52), dysphasic symptoms in 17% of auras (n=17). Twenty-six percent of patients had at least one aura out of three with one symptom lasting for more than one hour.

## Conclusions

This is the first study specifically focused on temporal aspects of migraine with aura. We provide data to suggest that aura symptoms may last longer than one hour in a relevant proportion of auras or migraine with aura patients. These findings will contribute to a better phenotypical framing of migraine with aura and may be of help in the review process of the international classification of headache disorders.

Written informed consent to publication was obtained from the patient(s).

## Conflict of interest

None.
